# The effect of phototherapy treatment on serum melatonin levels in term newborns

**DOI:** 10.55730/1300-0144.5816

**Published:** 2024-01-05

**Authors:** Ali BÜLBÜL, İlkay ÖZMERAL ODABAŞI, Duygu BESNİLİ AÇAR, Semra TİRYAKİ DEMİR

**Affiliations:** 1 Department of Neonatology, Şişli Hamidiye Etfal Education and Research Hospital, University of Health Science, İstanbul, Turkiye; 2 Department of Neonatology, Gaziosmanpaşa Education and Research Hospital, University of Health Science, İstanbul, Turkiye; 3 Department of Ophthalmology, Şişli Hamidiye Etfal Education and Research Hospital, University of Health Science, İstanbul, Turkiye

**Keywords:** Phototherapy, newborn, melatonin

## Abstract

**Background/aim:**

The aim of this study was to investigate the effect of phototherapy treatment on serum melatonin levels in term newborn infants.

**Material and methods:**

This study was planned as a single-center, prospective, observational, case-control study. Term infants (gestation week ≥37 weeks) who received at least 6 h of phototherapy due to jaundice constitute the phototherapy group, while the term infants without jaundice and who were exclusively breastfed constitute the control group. Melatonin levels were examined by taking blood samples from babies in both groups at 02:00 at night. Melatonin values were compared between groups. The effect of phototherapy on serum melatonin levels was investigated. The relationship between the duration of phototherapy and maximum serum bilirubin values on melatonin values was investigated.

**Results:**

Seventy term infants (64.3% girls) were included in the study. Mean gestational week was 38.3 ± 1.1 weeks, mean birth weight was 3295 ± 434 g. There was no statistically significant difference between the phototherapy group and the control group in terms of sex, type of delivery, gestational week, birth weight, height, and head circumference (all p > 0.05). Serum melatonin level was 20.3 ± 5.9 pg/mL (range: 8.7–36.6 pg/mL) in the phototherapy group and 19.9 ± 4.38 pg/mL (range: 9.9–26.3 pg/mL) in the control group. There was no significant difference between the two groups in terms of serum melatonin levels (p = 0.155). The mean total bilirubin value was 17.65 ± 1.48 mg/dL, and the average duration of phototherapy application was 13.94 ± 7.64 h in the babies in the phototherapy group. No correlation was found between the duration of phototherapy application and serum melatonin levels (p = 0.791).

**Conclusion:**

It was determined that there was no significant difference in serum melatonin levels in term newborn babies who received phototherapy for at least 6 h due to jaundice. No correlation was found between the duration of phototherapy application and the serum melatonin level of the maximum bilirubin values.

## 1. Introduction

The disease that most frequently requires hospitalization in the neonatal period is known as hyperbilirubinemia. At least two-thirds of newborns develop hyperbilirubinemia in the first week of life [1]. Although phototherapy is the oldest and most reliable treatment method for hyperbilirubinemia, many complications such as dehydration, fever, skin rashes, bronze baby syndrome, watery stools, and hypocalcemia have been reported [2–5]. It has been shown that hypocalcemia secondary to phototherapy may be caused by an increase in urinary calcium excretion and inhibition of melatonin secretion [6,7].

Melatonin (N-acetyl-5-methoxytryptamine) is a neurohormone secreted by the pineal gland, which has an effective protection against reactive oxygen species (ROS). It is usually secreted in the dark and its secretion is suppressed by light. The light cycle plays an important role in regulating the circadian rhythm. Melatonin is programmed to be synthesized at night by the suprachiasmatic nucleus in sync with the light-dark cycle via the retino-hypothalamic pathway [8–10]. The circadian rhythm of melatonin and the influence of human sleep have been extensively studied by many authors [11,12]. However, the importance of melatonin in the regulation of circadian rhythms in newborn babies who have undergone phototherapy has not been fully elucidated [13,14].

Studies on melatonin levels and the use of exogenous melatonin in the neonatal period are often associated with hypoxic ischemic encephalopathy and neonatal sepsis [15–18]. There are also studies related to the use of melatonin in the prevention of oxidative damage-related conditions such as retinopathy of prematurity, chronic lung injury, and necrotizing enterocolitis [19,20].

In this study, it was aimed to compare the serum melatonin levels of babies who received phototherapy due to hyperbilirubinemia and healthy term babies who did not receive phototherapy. We hypothesized that phototherapy may alter melatonin levels because of intense light exposure in infants treated with phototherapy.

## 2. Materials and methods

This study was planned as a single-center, prospective, observational, case-control study. The phototherapy group consisted of term infants (gestation week ≥37 weeks) who were hospitalized in the neonatal intensive care unit of our hospital due to hyperbilirubinemia, had no additional health problems, and were fed only with breast milk. Healthy infants born in our hospital during the same period and followed up with their mothers and fed only with breast milk constituted the control group.

Babies with a gestational week of <37 weeks, a pH of <7.2 in umbilical blood gas at birth, a history of smoking or alcohol use in their mothers, or cerebral anomalies were not included in the study.

### 2.1. Study design

Babies who received phototherapy for jaundice constituted the phototherapy group, while those without jaundice and who did not receive any treatment constituted the control group. The demographic characteristics of the babies in both groups included in the study (sex, gestational week, postnatal age, birth weight, birth length, birth head circumference, duration of phototherapy until the study included, and bilirubin levels) were recorded in the study form. The serum bilirubin level limit for phototherapy treatment was determined according to the American Academy of Pediatrics 2004 criteria [21]. Serum bilirubin value was measured using the spectrophotometric method (Roche Hitachi Cobas C 501). Phototherapy was applied with a device (Overhead Phototherapy System^®^, NOVOS, Turkiye) that emits waves at a wavelength of 440–460 nm (blue LED).

#### 2.1.1. Properties of ambient light

There are wall controls for ambient lighting in our clinic, allowing adjustment in three modes: daytime, nighttime, and off settings. During the study, the light was in nighttime mode (100–200 lux). A portable lamp (600 lux) was used during the blood collection procedure. Babies in the control group stayed with their mothers, and the ambient light in the mother’s room was 6–10 lux at night. A portable lamp (600 lux) was used for blood collection.

It was planned to take blood samples from the babies in both groups at 02:00 at night. Babies in the phototherapy group were included in the study if they had received at least 6 h of phototherapy before blood sampling, while those who received less than 6 h of phototherapy were excluded. Two millilitres of venous blood sample was taken from the babies in the phototherapy and control groups at 02:00 at night into a gel tube (BD Vacutainer^®^, UK). The blood sample taken was wrapped in aluminum foil to prevent contact with light and was centrifuged within 15 min and stored at −80 degrees. When the target number of cases was reached, the level of melatonin from the collected serum samples was examined. Serum melatonin level was studied with the enzyme-linked immuno sorbent assay (ELISA) method using an Elabscience kit (EL-H2016-Human Melatonin). According to the manufacturer the intra- and interassay coefficients of variation were 5.20%–5.45% in the range of 45.9–400.2 pg/mL and 4.28%–5.49% in the range of 43.5–418.6 pg/mL, respectively. The mean serum, EDTA plasma, and cell culture media recovery of melatonin was 102%, 98%, 96%, respectively, and the sensitivity of the assay was 9.38 pg/mL.

In the study, the effect of phototherapy application on serum melatonin levels was examined. In addition, the relationship between phototherapy duration and maximum serum bilirubin values with melatonin values was investigated.

### 2.2. Sample size

The sample size was calculated with the G*Power v. 3.1.6 program, at an alpha significance level of 0.05 with 95% power, considering the large effect size (effect size = 0.8) to indicate a difference between the groups. It was determined that a total of 70 cases were required, with 35 cases for each group.

### 2.3. Statistics

SPSS package program v. 20.0 was used for statistical analysis in the evaluation of the patient data. Descriptive statistics were given as numbers and percentages for categorical variables, and as mean, standard deviation, minimum, maximum, and median for numerical variables. Comparison of numerical variables in the independent group was evaluated with Student’s t-test in the normal distribution condition and with the Mann–Whitney U test in the absence of the normal distribution condition. Relationships between numerical variables were assessed with Pearson correlation analysis in the presence of parametric test condition and with Spearman correlation analysis in the absence of parametric test condition. Statistical alpha significance level was accepted as p < 0.05.

### 2.4. Ethical issues

The study was approved by the Şişli Hamidiye Etfal Hospital Ethics Committee (1023–2018). Written informed consent was obtained from the parents of all infants included in the study. The study was conducted in accordance with the principles stated in the Declaration of Helsinki.

## 3. Results

The study was completed with a total of 70 infants, 35 of which were in the phototherapy group and 35 in the control group. The distribution of demographic characteristics of both groups in the study is shown in Table. The mean birth weight of the babies was 3295 ± 434 g (range: 2380–4560 g), the mean height was 49.7 ± 2.2 cm (range: 44–56 cm), and the mean head circumference was 34.5 ± 1.3 cm (range: 32.5–35.5 cm). There was no statistically significant difference between the phototherapy group and the control group in terms of sex, type of delivery, gestational week, birth weight, height, and head circumference (p > 0.05).

The serum melatonin levels of the babies included in the study are shown in Table. The mean melatonin level of 70 newborns included in the study was 20.1 ± 5.2 pg/mL (range: 8.7–36.6 pg/mL). There was no significant difference between the two groups in terms of serum melatonin levels (Figure). The mean total bilirubin value of the babies in the phototherapy group was 17.65 ± 1.48 mg/dL (range: 15.2–21 mg/dL). An average of 13.94 ± 7.64 h (range: 8–40 h) of phototherapy was applied to the babies in the phototherapy group. No correlation was found between the duration of phototherapy and serum melatonin level (p = 0.791).

## 4. Discussion

Melatonin is synthesized from the amino acid tryptophan as a precursor and is released from the pineal gland. The primary regulation of melatonin, which is released with the circadian rhythm, is provided by the light/dark cycle [22]. In adult human studies, it has been shown that a blue/green LED (light emitting diodes, wavelength 485–510 nm) light source reduces the amount of nocturnal salivary melatonin and disrupts the circadian rhythm [23]. It has been reported that the nighttime melatonin level is suppressed by approximately 50% in adults exposed to this light for 2 h [23]. Studies on melatonin levels in the neonatal period are very few. It was determined that the level of 6-hydroxy-melatonin-sulfate (6OHMS), a melatonin metabolite, was low in newborn babies who stayed in the incubator for at least 2 days. This is since the incubator creates an electromagnetic field (EMF) and reduces melatonin release [24].

Phototherapy, which was started to be used in the treatment of neonatal hyperbilirubinemia about 60 years ago, is the oldest known treatment method of hyperbilirubinemia. The efficacy of phototherapy for neonatal hyperbilirubinemia was first reported in 1958 by Cremer et al. [25]. Despite its ongoing use since 1958, data on the relationship of phototherapy with melatonin are limited. Melatonin secretion is dependent on pinealocyte cells and their sensitivity to light. Thanks to this sensitivity, while melanocytes secrete melatonin in the dark, oscillation with light is prevented. The secretion of melatonin peaks between 02:00 and 04:00 hours, with its concentration in the blood increasing 3–10 times^1^. It has been reported that the melatonin circadian rhythm emerges and continues at the end of the neonatal period [26]. There are limited studies evaluating the relationship between phototherapy and melatonin. Hakanson et al., who conducted one of the first studies on this subject, reported that the decrease in serum calcium levels in experimental mice using white light may be related to melatonin. They suggested occiput protection, inhibition of corticosterone synthesis, and exogenous melatonin intake to prevent this metabolic disorder [27]. About 40 years after this historically significant study, Asghar et al. reported that there was a significant decrease in serum calcium level in newborns who received phototherapy without wearing a headgear compared to newborns who used headgear during phototherapy [5,28].

There are very few studies on melatonin levels in the neonatal period. In these studies, gestational week and serum melatonin evaluation time show differences. In the study of Biran et al. in which they compared plasma melatonin concentrations in preterm and term newborns, the mean serum melatonin value on the postnatal 3rd day was found to be 8 pg/mL (range: 7–21 pg/mL) in 86 babies in the group whose gestational week was between 34 weeks and 41 weeks 6/7 [29]. Muñoz-Hoyos et al. studied melatonin levels in the first week of life. They found the mean serum melatonin level to be 96.28 ± 30.0 pg/mL on the postnatal 3rd day and 109.48 ± 24.0 pg/mL on the postnatal 7th day in 17 newborns with a mean gestational week of 33.9 weeks (range: 28–40 weeks) and a birth weight of >1500 g [30]. In our study, melatonin levels were found to be lower than the values reported in both the phototherapy group and the healthy control group. It was thought that the difference in reported melatonin levels between studies may be related to the race, gestational age, birth weight, time of blood collection during the day, and heterogeneity between the cases in the studies. In addition, in the study of Muñoz-Hoyos et al., it was determined that serum melatonin samples were taken at 09:00 [30]. Considering the circadian rhythm of the serum melatonin level, it was thought that the difference in the results might be related to the collection time of the samples among the cases included in the studies. Vigneshwar et al. investigated the relationship between phototherapy and serum calcium and serum melatonin levels in 89 newborns with mean total serum bilirubin levels of 14.1 ± 2.8 mg/dL. Similar to our study, they reported that there was no statistically significant decrease in median serum melatonin levels after phototherapy [31]. Chen et al. investigated the effect of phototherapy on the expression of circadian genes and plasma melatonin levels in peripheral blood mononuclear cells in 32 newborns. Serum melatonin levels were evaluated before and after phototherapy in infants who received phototherapy for 24 h, and they reported a statistically significant decrease in melatonin levels after treatment, unlike our result [32]. In this study, different from our study, infants who received phototherapy for 24 h were included in the study. In our study, the mean phototherapy time at the time of collection of samples was 13.37 ± 7.84 h. In our study, we did not find a correlation between the duration of phototherapy and serum melatonin levels. In other studies, the decrease in serum melatonin levels may be due to longer-term phototherapy applications. Jaldo-Alba et al. divided the cases into three groups which were 24, 48 and 72 h according to the duration of phototherapy and evaluated serum melatonin levels before and after phototherapy. Unlike other studies, they found a significant increase in serum melatonin levels after phototherapy. They interpreted this situation as closing the eyes of babies during phototherapy causes light deprivation and may increase melatonin levels [33].

In a study conducted on 14 adult volunteers in intensive care unit, it was reported that using eye mask patches and earplugs increased the level of urinary 6-sulfatoxymelatonin [34]. Eye masks for babies are used during phototherapy. This may be the reason why melatonin levels were not different in the phototherapy group in our study.

The strengths of our study are that it is prospective and case controlled. However, measuring the melatonin metabolite level in the urine samples of infants would have increased the efficiency of the study. Including infants with a longer duration of phototherapy by forming a subgroup would have increased the strength of our study.

In conclusion, in our study, it was determined that there was no significant difference in serum melatonin levels in infants who received phototherapy for at least 6 h compared to healthy controls. Although the level of melatonin in the neonatal period is associated with many factors such as gestational week, weight, exposure to light, and phototherapy application, data on this subject is limited. The use of an eye mask during phototherapy in term newborn babies has been found to be an effective practice in ensuring that melatonin levels remain within normal limits. The fact that different results have been obtained regarding the relationship between phototherapy and serum melatonin levels suggests that there is a need for standardized, large series studies on this subject.

## Figures and Tables

**Figure:**
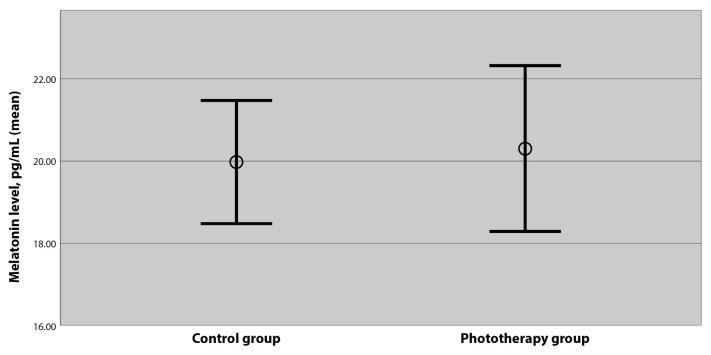
The comparison of serum melatonin levels of babies who received at least 6 hours phototherapy due to hyperbilirubinemia and healthy term babies (p: 0.155).

**Table:** Distribution of demographic characteristics of infants included in the study by groups.

	All patients (n:70)	Phototherapy group (n:35)	Control group (n:35)	p
*Weight (g)	3295 ± 434	3270 ± 396.5	3321 ± 474	0.627Φ
*Height (cm)	49.7 ± 2.2	49.7 ± 2.1	49.8 ± 2.3	0.874Φ
*Head circumference (cm)	34.5 ± 1.3	34.6 ± 1.4	35.1 ± 1.1	0.117Φ
Gestation time (weeks)	38.3 ± 1.1	38.5 ± 1.1	38.1 ± 1	0.184Φ
Sex n(%)				
Female	45 (64.3)	24 (68.5)	21 (60)	0.309£
Male	25 (35.7)	11 (31.4)	14 (40)	
Type of birth n (%)				
Normal spontaneous birth	32 (45.7)	19 (54.3)	13 (37.2)	0.230£
Cesarean section	38 (54.3)	16 (45.7)	22 (62.8)	
Melatonin level, pg/mL (range)	20.1 ± 5.2 (8.8–36.6)	20.3 ± 5.9 (8.7–36.6)	19.9 ± 4.38 (9.9–26.3)	0.155Φ

* At birth

Φ Independent samples t-test

£ Chi-squared test

## Footnotes

1 Arendt J, Aulinas A. Physiology of the Pineal Gland and Melatonin. In: Feingold KR, Anawalt B, Blackman MR et al., editors. Endotext (2022). South Dartmouth (MA) [online]. Website https://www.ncbi.nlm.nih.gov/books/NBK550972 [accessed 10/01/2023].
